# A case of insulin-like growth factor 2-producing gastrointestinal stromal tumor with severe hypoglycemia

**DOI:** 10.1186/s12902-020-0529-2

**Published:** 2020-05-11

**Authors:** Haruka Yamasaki, Ayako Itawaki, Miwa Morita, Hitomi Miyake, Masahiro Yamamoto, Hiroki Sonoyama, Sayuri Tanaka, Masakazu Notsu, Mika Yamauchi, Yusuke Fujii, Noriyoshi Ishikawa, Izumi Fukuda, Shunji Ishihara, Keizo Kanasaki

**Affiliations:** 1 Department of Internal Medicine 1, Shimane University Faculty of Medicine, 89-1, Enya-cho, Izumo, Shimane 693-8501 Japan; 2 Department of Internal Medicine 2, Shimane University Faculty of Medicine, 89-1, Enya-cho, Izumo, Shimane 693-8501 Japan; 3 Department of Digestive and General Surgery, Shimane University Faculty of Medicine, 89-1, Enya-cho, Izumo, Shimane 693-8501 Japan; 4 Department of surgical pathology, Shimane University Faculty of Medicine, 89-1, Enya-cho, Izumo, Shimane 693-8501 Japan; 5 Division of diabetes, Endocrinology and Metabolism, Department of Medicine, Nippon Medical School, 1-1-15, sendagi, bunkyo-ku, 113-8603 Japan; 6 Division of Anticipatory Molecular Food Science and Technology, Medical Research Institute, Kanazawa Medical University, Uchinada, Ishikawa 920-0293 Japan

**Keywords:** NICTH, GIST, Hypoglycemia, Big-IGF2

## Abstract

**Background:**

Non-islet cell tumor hypoglycemia (NICTH) is a rare paraneoplastic syndrome that secretes incompletely processed high molecular weight insulin growth factor 2 (big-IGF2), which results in stimulation of the insulin receptor and subsequently induces hypoglycemia. Gastrointestinal stromal tumor (GIST) is a common intestinal mesenchymal neoplasm of the gastrointestinal tract. The most frequent site of GIST is the stomach; NICTH induced by IGF2-producing stomach GISTs is rare.

**Case presentation:**

An 84-year-old man was admitted to the hospital due to impaired consciousness (JCS II-10) in the morning. At the time of admission, his serum glucose was 44 mg/dL; his consciousness was restored with 20 ml of 50% glucose. To avoid hypoglycemia, a continuous intravenous infusion of glucose as well as dietary intervention was required. At the time of hypoglycemia, the levels of insulin and C-peptide were suppressed. Additionally, IGF1 levels were below the normal range. Abdominal computed tomography revealed that he had a large lobulated mass (116 × 70 × 72 mm) around the gastric corpus. Pathological analysis of biopsy specimens identified disarray of spindle cells and positivity for c-kit as well as strong positivity for DOG-1. Further analysis revealed high levels of Ki-67 (Mib-1 index: 15.5%) and mitotic index (7/50HPF); the tumor was diagnosed as high-risk GIST, and complete surgical resection was performed. Hypoglycemia resolved immediately after tumor resection. The resected tumor specimen was positive for IGF2 staining, and big-IGF2 (11–18 kDa) was detected in preoperative serum and tumor samples; the patient was diagnosed with NICTH due to an IGF2-producing tumor.

**Conclusions:**

NICTH is rare in GIST of the stomach; however, the large GIST could produce big-IGF2 and subsequently cause severe hypoglycemia, requiring prompt evaluation and complete tumor resection.

## Background

Non-islet cell tumor hypoglycemia (NICTH) is a rare paraneoplastic syndrome and has an estimated incidence of approximately one per million person-years [1]. NICTH is induced by incomplete processing of tumor-secreted high molecular weight insulin-like growth factor2 (IGF2), known as big-IGF2 or pro-IGF2E [68–88] [2] [3], which stimulates the insulin receptor and glucose utilization and subsequently induces hypoglycemia [1]. Gastrointestinal stromal tumor (GIST) is a common intestinal mesenchymal neoplasm of the gastrointestinal tract that is mainly characterized by the overexpression of receptor tyrosine kinase KIT [4] [5]. Although the most common site of GIST is the stomach (39 to 72.3% [6] [7] [8]), NICTH induced by IGF2-producing GIST of the stomach has shown to be rare [2].

Here, we report a NICTH case induced by IGF2-producing primary gastric GIST.

## Case presentation

An 84-year-old man was admitted to an affiliated hospital due to an impaired level of consciousness (JCS II-10) in the morning.

He experienced rotatory vertigo, weakness and dizziness without any particular cause form 1 week before the admission to that hospital. Three days prior to the admission, he had fainted and fallen to the floor in the early morning. At the time of admission in that hospital, his serum glucose was 44 mg/dL, and after infusion of 20 ml of 50% glucose solution, his consciousness was restored. Blood pressure was 184/100 mmHg, and heart rate was 65/min. He did not have fever, his respiratory rate was 16/min, and SpO_2_ was 95% at ambient air. Other physical examinations were unremarkable. Abdominal computed tomography (CT) in the emergency unit demonstrated a large lobulated mass around the gastric corpus ~ φ120 mm. For further analysis, he was transferred to our hospital.

When transferring to our hospital, he was alert with continuous intravenous infusion of glucose. His blood pressure and heart rate were also in the normal range. He had a 2-year history of hypertension that was treated with olmesartan. He was prescribed atorvastatin, tamsulosin, and brinzolamide/timolol maleate eye drops for dyslipidemia, benign prostate hyperplasia, and glaucoma, respectively. He had taken sennoside for constipation as needed. He did not have a history of diabetes, although both his older sister and brother did. He has been followed up and monitored for intraductal papillary mucinous neoplasm (IPMN) with abdominal CT at an affiliated hospital.

**Fig. 1:**
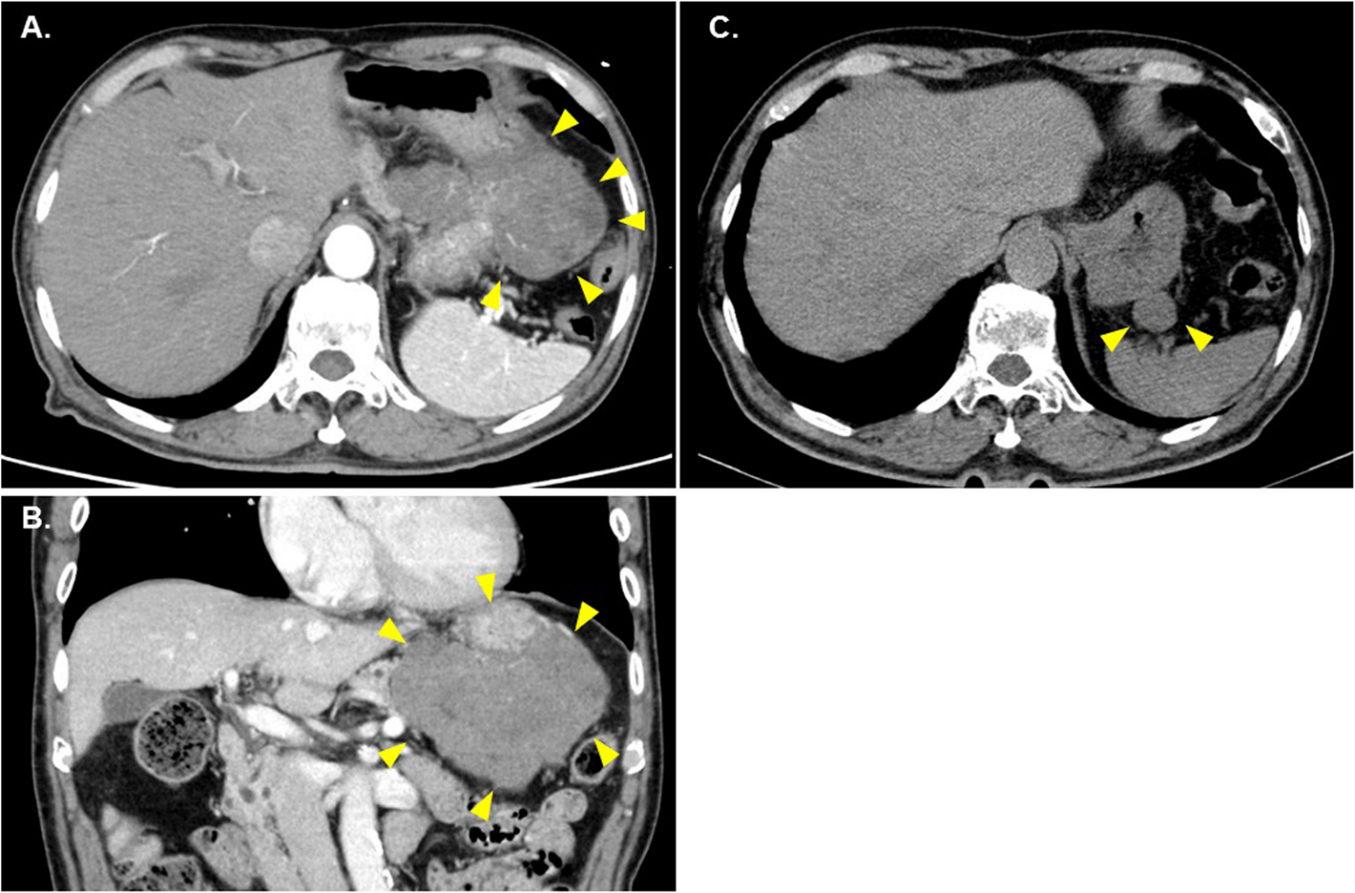
CT scan of the abdomen. **a** and **b**; at the time of admission. **c**; one and a half years ago of admission

After admission to our hospital, he required a continuous infusion of glucose (4.2 g/h), a diet of 1800 kcal per day and supplementary meals of 380 kcal before sleep to avoid hypoglycemia. Insulin and C-peptide were suppressed at the time of hypoglycemia (Table 1). IGF1 was below the normal range (22 ng/mL) (Table 1). Hypothalamic-pituitary dysfunction was not likely the cause of his hypoglycemia (Table 1). Contrasted abdominal CT revealed a large lobulated mass of 116 × 70 × 72 mm with central necrosis and heterogeneous enhancement around the gastric corpus (Fig. 1 a and b). A retrospectively unclassified ~ φ20 mm mass was found in the same location on a CT (Fig. 1c) for follow-up of regular IPMN (1.5 years ago), indicating a rapid increase in the tumor mass. NICTH was suspected due to fasting hypoglycemia, low IGF-I, suppression of insulin secretion and a large tumor.

**Table 1:** Laboratory findings on admission

Variable	Reference Range (Affiliated Hospital/ This Hospital)	On Admission Affiliated Hospital	Next Day Admission Affiliated Hospital	2 Days after Admission Affiliated Hospital	On Admission This Hospital	16 Days after Admission This Hospital
Blood						
Hematocrit	36–48/40.7–50.1 (%)	39.2			41	
Hemoglobin	13.5–17.6/13.7–16.8 (g/dL)	13.3			13.5	
White-cell- count	3500-9800/3300–8600 (/μL)	5300			5360	
Platelet count	13.0–36.9/15.8–34.8 (× 10^4^/μL)	9.8			12.2	
Albumin	3.9–4.9/4.1–5.1 (g/dL)	3.8			3.9	
Total Bilirubin	0.2–1.2/0.4–1.5 (mg/dL)	0.8			0.7	
AST	8–38/13–30 (U/L)	28			30	
ALT	4–44/10–42 (U/L)	13			16	
LDH	106–211/124–222 (U/L)	257			253	
ALP	104–338/106–322 (U/L)	226			250	
γ-GTP	18–66/13–64 (U/L)	20			21	
BUN	8.0–20.0/8.0–20.0 (mg/dL)	12.2			14.2	
Creatinine	0.40–1.10/0.65–1.07 (mg/dL)	0.57			0.58	
eGFR	(mL/min/1.73m^2^)	128			98.7	
Sodium	135–147/138–145 (mmol/L)	141			142	
Potassium	3.3–4.8/3.6–4.8 (mmol/L)	3.3			3.9	
Chlor	98–108/101–108 (mmol/L)	108			105	
HbA1c	5.0–6.2/4.9–6.0 (%)	5.7			5.9	
Plasma glucose	70–110/73–109 (mg/dL)	44	47	40	147	57
Insulin	1.09–17.02 /5.0–20.0 (μU/mL)	0.2				< 2.0
C peptide	0.8–2.5 (ng/mL)					< 0.2
ACTH	7.2–63.3/7.7–63.1 (pg/mL)		53.1			
Cortisol	4.5–21.1/2–18 (μg/dL)		17.3			9
Growth hormone	−2.47/−3 (ng/mL)			0.34		4.2
Insulin growth factor 1	84–177^a^ (ng/mL)			22		21
Adrenalin	−0.10/−0.17 (ng/mL)			0.11		0.06
Noradrenalin	0.10–0.50/0.15–0.57 (ng/mL)			0.27		0.26
Dopamine	−0.03/−0.03 (ng/mL)			≤ 0.01		≤ 0.02
Glucagon	71–174 pg/mL (pg/mL)					154
Anti-insulin antibody	−0.4 (%)		<0.4			

Glucagon and IGF1 were measured by radioimmunoassay (RIA); ACTH, cortisol, and growth hormone were measured by chemiluminescent enzyme immunoassay (CLEIA); adrenalin, noradrenalin, and dopamine were measured by High Performance Liquid Chromatography (HPLC)

^a^Reference range of Insulin growth factor 1 (IGF1) for 77 years old male. The reference range of IGF1 is differ by age and sex, only up to 77 years old

**Fig. 2:**
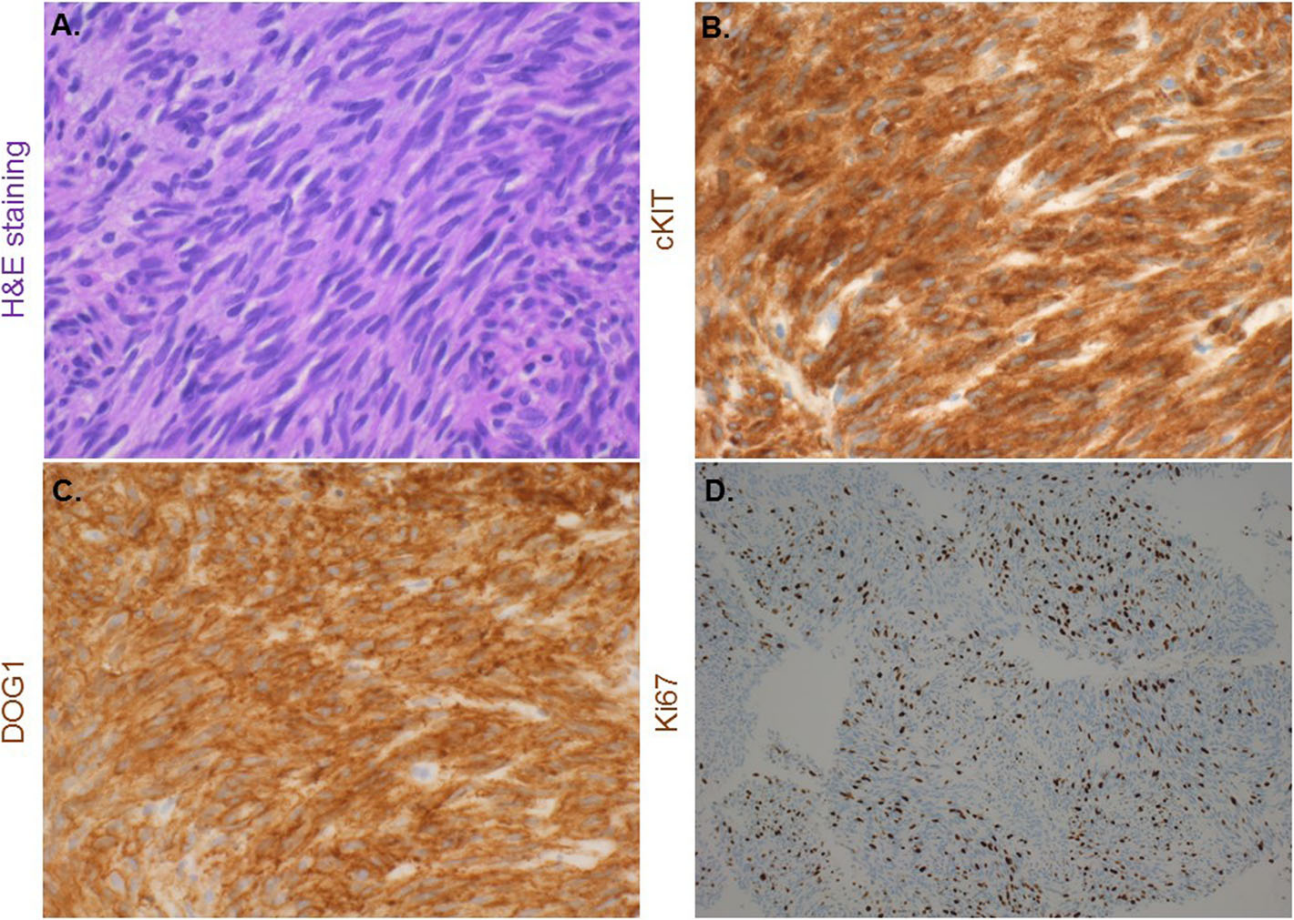
Pathological findings of endoscopic ultrasound-guided fine-needle aspiration biopsy. **a**. Hematoxylin-eosin (HE) staining shows hyperplastic spindle cell. (X400). **b**. Immunostaining for c-KIT is diffuse positive in tumor cytoplasm. (X400). **c**. Immunostaining for Discovered on DOG-1 is diffuse strongly positive in tumor cytoplasm. (X400). **d**. Immunostaining for ki-67 is positive in nucleus. (X400)

Pathological analysis of an endoscopic ultrasound-guided fine-needle aspiration biopsy specimen identified a disarray of spindle cells and the presence of c-kit positivity as well as strong positivity for DOG-1, confirming GIST histology (Fig. 2). Further analysis revealed high levels of Ki-67 (Mib-1 index: 15.5%) and mitotic index (7/50HPF). The tumor was diagnosed as high-risk GIST, and complete surgical resection was performed on hospital day 26. Hypoglycemia was disappeared immediately after the operation. Beginning the next day, neither glucose infusion nor supplementary meals before sleeping were required to maintain the blood glucose levels of the patient.

**Fig. 3:**
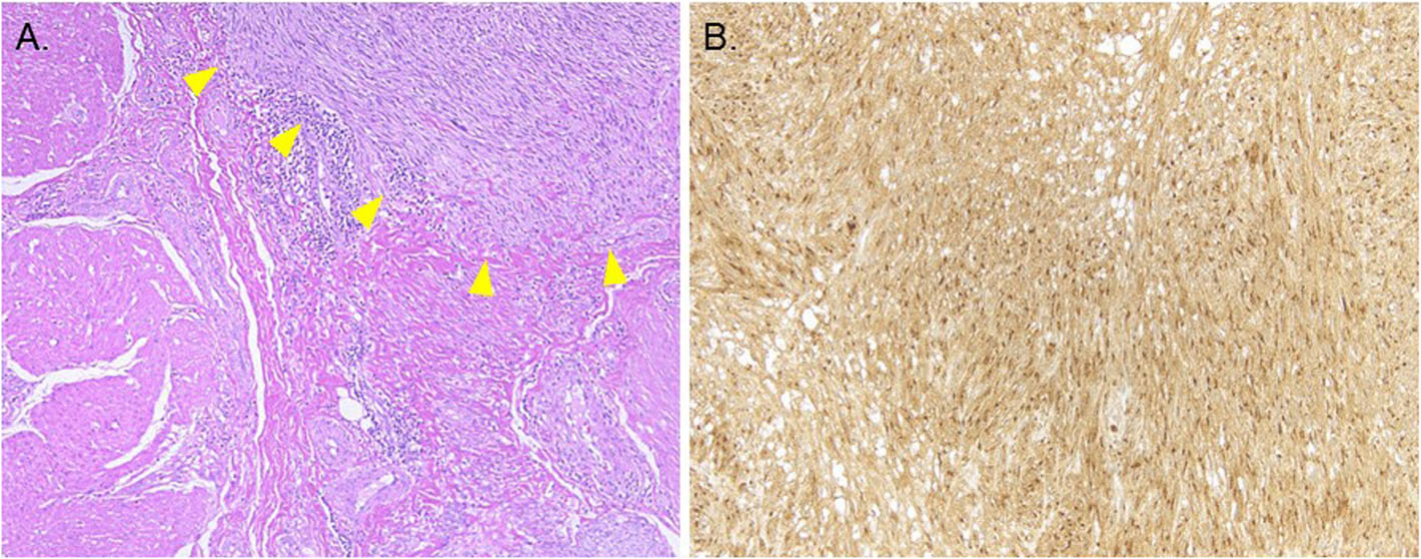
Pathological findings of the resected specimen. **a**. Hematoxylin-eosin (HE) staining shows that tumor is primary muscular coat of gastric. (X100). **b**. Immunostaining for IGF2 is diffuse positive in tumor cytoplasm. (X100)

**Fig. 4:**
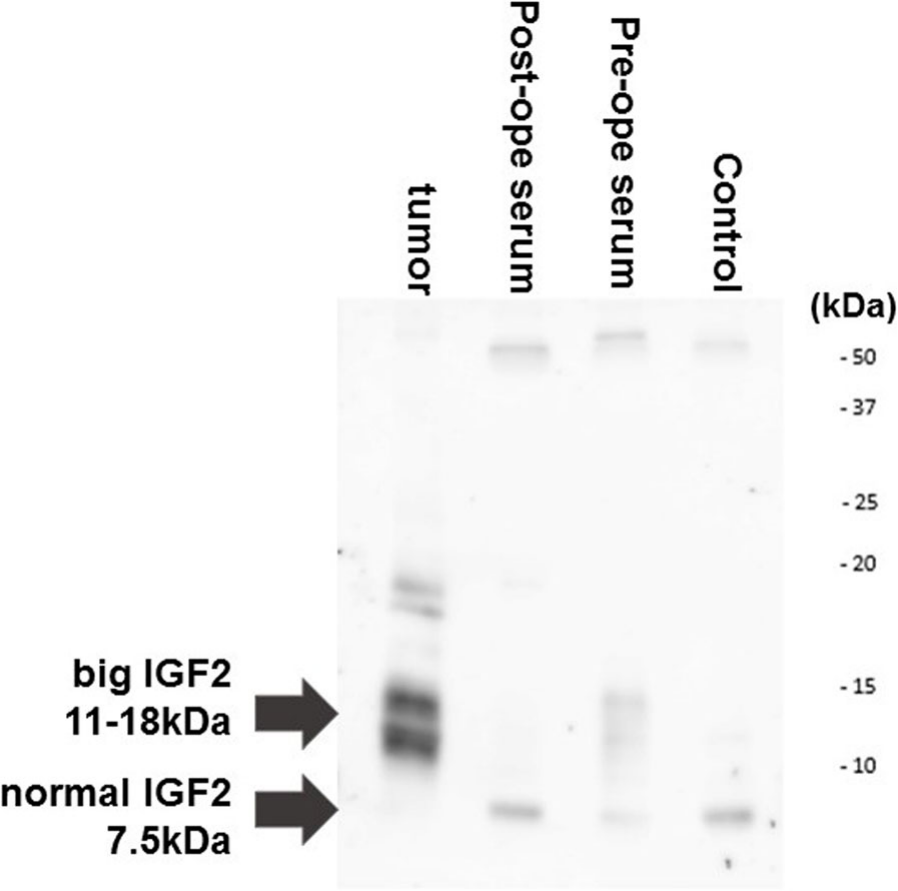
Western immunoblot analysis of serum and tumor insulin growth factor 2 (IGF2). Big IGF2 (11–18kDA) was detected only in the pre-operative serum and tumor samples. Acid-ethanol extracted serum samples and tumor sample were performed a Western immunoblotting using clone S1F2, Merck Millipore, Japan for primary antibody

There was no obvious metastasis of the tumor. The resected specimen was positive for IGF2 staining (Fig. 3). Histological examination revealed the stomach-origin IGF2-producing GIST. When evaluated 1 week after the operation, the level of IGF2 was decreased (Table 2). IGF2/IGF1 remained higher; IGF1 levels were elevated to 64 ng/mL at 3 weeks after the operation. In preoperative serum and tumor samples, big-IGF2 (11–18 kDa) was detected, and such aberrant IGF2 disappeared (Fig. 4). A final diagnosis was made as NICTH due to IGF2 production.

**Table 2:** IGF2 and IGF1 levels at pre and postoperative

	Pre operation	One week after operation
IGF1 (ng/mL)	21	39
IGF2 (ng/mL)	772	492
IGF2/IGF1	33.6	12.6

*IGF1* Insulin growth factor 1, *IGF2* Insulin growth factor 2

Normal Range: IGF1 (84–177^a^), IGF2 (374–804), IGF2/IGF1 (3.3–0.2)

^a^Normal range of IGF-1 for 77 years old man. The reference range of IGF1 is differ by age and sex, only up to 77 years old

Since then, he has been regularly monitored by CT in our hospital, and is free from relapse of tumor or hypoglycemia for 2.5 years.

## Discussion and conclusions

NICTH is a rare paraneoplastic syndrome in which a tumor secretes high molecular weight IGF2, causing hypoglycemia [1]. The major effects of IGF2, a polypeptide hormone, are growth and cell differentiation promotion in the fetal period; IGF2 also has a similar action as insulin, yet its biological activity is low (approximately 14% of that of insulin) [9].

The *Igf2* gene is located on the short arm of chromosome 11 (11q15.5). The transcriptional product of the *Igf2* gene is translated into a pre-pro-IGF2 polypeptide, and subsequently, posttranslational modification forms it into pro-IGF2. Pro-IGF2 is processed into mature IGF2, which has a molecular weight of approximately 7.5 kDa. However, in most IGF2-producing NICTH, incomplete processing occurs, and one part of the E chain (C-terminal lesion of Pro-IGF2) remains; high molecular weight IGF2, or so-called big-IGF2 (molecular weight: 11–18 kDa) [10], is then detected in blood and tumors. Although the IHC stain for normal IGF2 of tumor is strong positive, a normal IGF2 was not detected in Western blotting in Fig. 4. A high molecular weight IGF2, known as a big-IGF2 or pro-IGF-2E (68–88), contained same amino-acid sequence with normal IGF2 region, therefore it is impossible to distinguish large molecule IGF2 from normal IGF2 by IHC. Western blot analysis for tumor sample did not display normal IGF2 (7.5 kDa) (if any, very tiny levels) but with very high levels of big IGF2, suggesting that majority of IGF2 produced in tumor is big-IGF2. The big-IGF2-IGF binding protein-3 (IGFBP-3) complex does not form a trimer with an acid labile subunit (ALS); therefore, it is easily mobilized to the tissue where it exerts biological activity. Once the tumor was completely resected and hypoglycemia disappeared, such aberrant IGF2 disappeared from the blood, and the normal mature IGF2 level could increase [11]. In our case, 11–18 kDa big-IGF2 was found in the serum and tissues before surgery by Western blot examination and disappeared after surgery. Tumors of 12 cm or greater were associated with significantly higher blood big-IGF2 concentrations [12]. Since IGF2 displays mitogen [3], the induction of IGF2 may contribute to the malignant transformation of GIST. Our case had a large tumor size of 12 cm, the MIB-1 index was as high as 15.5%, exhibiting characteristics of a high-risk tumor, and big-IGF2 could contribute to malignant transformation.

Most IGF2-producing tumors are large, and 70% are φ10 cm or greater [13]. In our case, the tumor size was φ12 cm; the blood concentration of IGF2 was 722 ng/mL and remained in the normal range (374–804). Regarding IGF2 concentration, only approximately 1/3 of cases display high IGF2 blood levels in NICTH associated with IGF2-producing tumors [13]. Therefore, the IGF2/IGF1 ratio could be useful as a reference instead of the IGF2 value itself [14]. In our case, the preoperative IGF2/IGF1 ratio was as high as 33.6, consistent with an IGF2-producing tumor. One week after the operation, the IGF2/IGF1 ratio decreased but remained high, suggesting that the IGF1 level was not yet normalized. The reason why the restoration of IGF1 levels was not completed in our patients after operation was not clear yet. The big IGF2 is thought to suppress GH secretion and thereby reduces IGF1, therefore it could be possible that GH-IGF1 axis is not normalized yet at 1 week after operation. Also, we could not measure the levels of IGFBPs, by which IGFs levels and turn over were significantly modified [15]. Therefore, the potential contribution of IGFBPs level alteration before and after operation on the levels of IGF1 cannot be excluded. GIST is a common gastrointestinal mesenchymal tumor. The stomach is the most frequent site of GIST (39 to 72.3% [6] [7] [8]); IGF2-producing gastric GIST is rare [2]. Bodnar reported that 11 out of 288 NICTH cases were caused by GIST. Furthermore, out of the 11 NICTH cases caused by GIST, only 1 case was associated with primary gastric GIST [2]. More frequent expression of big-IGF2 has been observed in moderate and higher risk tumors compared to tumors with a lower risk classification [16]. The level of big-IGF2 in gastric GIST has been shown to be relatively lower than that of the small intestine/colon [16], indicating the reason why a lower frequency of NICTH is associated with gastric GIST. The details by which lower big-IGF2 levels occur in gastric GISTs have not yet been elucidated, and further analysis is required.

In conclusion, we report a case of NICTH with IGF2-producing primary gastric GIST. The frequency of NICTH associated with GIST of the stomach is rare; however, the large and high-risk GIST could produce big-IGF2 and subsequently cause severe hypoglycemia, requiring prompt evaluation, interpretation and complete tumor resection.

## Data Availability

Not applicable.

## Abbreviations

NICTH: Non-islet cell tumor hypoglycemia
IGF: Insulin growth factor
GIST: Gastrointestinal stromal tumor
CT: Computed tomography
IPMN: Intraductal papillary mucinous neoplasm
ACTH: Adrenocorticotropic hormone
GH: Growth hormone
